# Development and application of a risk prediction nomogram for sarcopenia one year after surgery in early-stage non-small cell lung cancer patients

**DOI:** 10.3389/fmed.2026.1903495

**Published:** 2026-08-19

**Authors:** JiaWei Zhang, Lun Li, Heng Chu, Zhe Zhang

**Affiliations:** 1 Department of Thoracic Surgery, Hunan Provincial Chest Hospital, Changsha, China; 2 The Affiliated Chest Hospital of Hunan Normal University, Changsha, China; 3 Department of Thoracic Surgery, Qingdao Municipal Hospital, Qingdao, China

**Keywords:** nomogram, non-small cell lung cancer, prediction model, sarcopenia, surgery

## Abstract

**Background:**

Postoperative sarcopenia is a prevalent complication in early-stage non-small cell lung cancer (NSCLC) patients, adversely affecting their prognosis. Accordingly, identifying individuals at risk of sarcopenia is crucial for appropriate assessment and treatment. This research sought to determine predictors of sarcopenia one year after minimally invasive pulmonary resection (post-sarcopenia) in early-stage NSCLC patients and to create and validate a clinical screening model for this condition.

**Methods:**

This study involved patients who had video-assisted thoracoscopic lobectomy or sublobectomy between January 2018 and December 2020, with chest computed tomography (CT) conducted at 1 year postoperatively. Post-sarcopenia was diagnosed by calculating the skeletal muscle index from the skeletal muscle area measured at the T12 vertebral level using cross-sectional CT scans. Subjects were randomly divided into training and validation sets. The training set underwent univariate and multivariate analyses to identify risk factors for post-sarcopenia and establish a risk score for predicting the development of post-sarcopenia. The risk score’s effectiveness was assessed through the area under the receiver operating characteristic curve (AUC), calibration, and decision curve analysis (DCA) in both cohorts.

**Results:**

The study included 251 patients (mean age 64.4 years; 63.75% female), divided into a training set (*n* = 175) and a validation set (*n* = 76). Six variables were identified as independent predictors and incorporated into the post-sarcopenia risk score: gender, body mass index, surgical method, prolonged air leak, C-reactive protein, and postoperative albumin. The risk score demonstrated good discriminatory ability for predicting post-sarcopenia, achieving an average AUC of 0.89 (95% CI: 0.84–0.94) in the training set and 0.84 (95% CI: 0.74–0.93) in the validation set, along with satisfactory calibration (all *p* > 0.05). The DCA demonstrated the net benefit of the risk score across various risk thresholds in both cohorts.

**Conclusion:**

The developed and validated post-sarcopenia risk score demonstrates strong predictive ability for identifying patients at risk of sarcopenia following minimally invasive pulmonary resection for early-stage NSCLC patients. This risk score can assist clinicians in enhancing post-sarcopenia screening, evaluating patient prognosis, and formulating targeted strategies.

## Introduction

Lung cancer is the most prevalent cancer globally and the leading cause of cancer-related mortality. Non-small cell lung cancer (NSCLC) is the predominant pathological type, accounting for approximately 80–85% of cases (1). Despite advances in NSCLC treatment that have improved, the 5-year postoperative survival rate remains under 75% (2).

Sarcopenia, marked by the progressive and generalized decline in skeletal muscle mass and strength, has emerged as a major concern (3). Currently, skeletal muscle area (SMA) measured by chest computed tomography (CT) images, standardized by the skeletal muscle index (SMI), can be used as a basis for diagnosing sarcopenia (4). In lung cancer patients, postoperative sarcopenia has been established as an important prognostic factor (5). Research indicates that sarcopenia is linked to negative outcomes such as postoperative complications, extended hospitalizations and decreased survival rates (6, 7). Specifically, the presence of sarcopenia may lead to greater challenges in recovery for lung cancer patients after surgery, affecting their overall survival rate and quality of life (8).

In clinical practice, early recognition and intervention for sarcopenia may improve the postoperative prognosis of lung cancer patients. For instance, nutritional interventions and tailored exercise programs can mitigate the effects of sarcopenia to some extent, thereby improving postoperative survival rates and quality of life (9). Therefore, when developing treatment plans for lung cancer patients, clinicians should consider assessing muscle quality and implementing corresponding measures to improve the patient’s overall health, ultimately aiming to enhance postoperative outcomes.

Nomograms, as a simple statistical prediction tool, have been widely applied in clinical diagnosis and prognosis (10). However, limited research has focused on models for predicting the risk of sarcopenia one year after minimally invasive pulmonary resection in early-stage NSCLC patients. The present study sought to develop nomograms to model postoperative sarcopenia incidence in NSCLC patients, thereby identifying high-risk groups and providing new insights for sarcopenia screening in postoperative NSCLC cases.

## Method

### Patients

**Figure 1:**
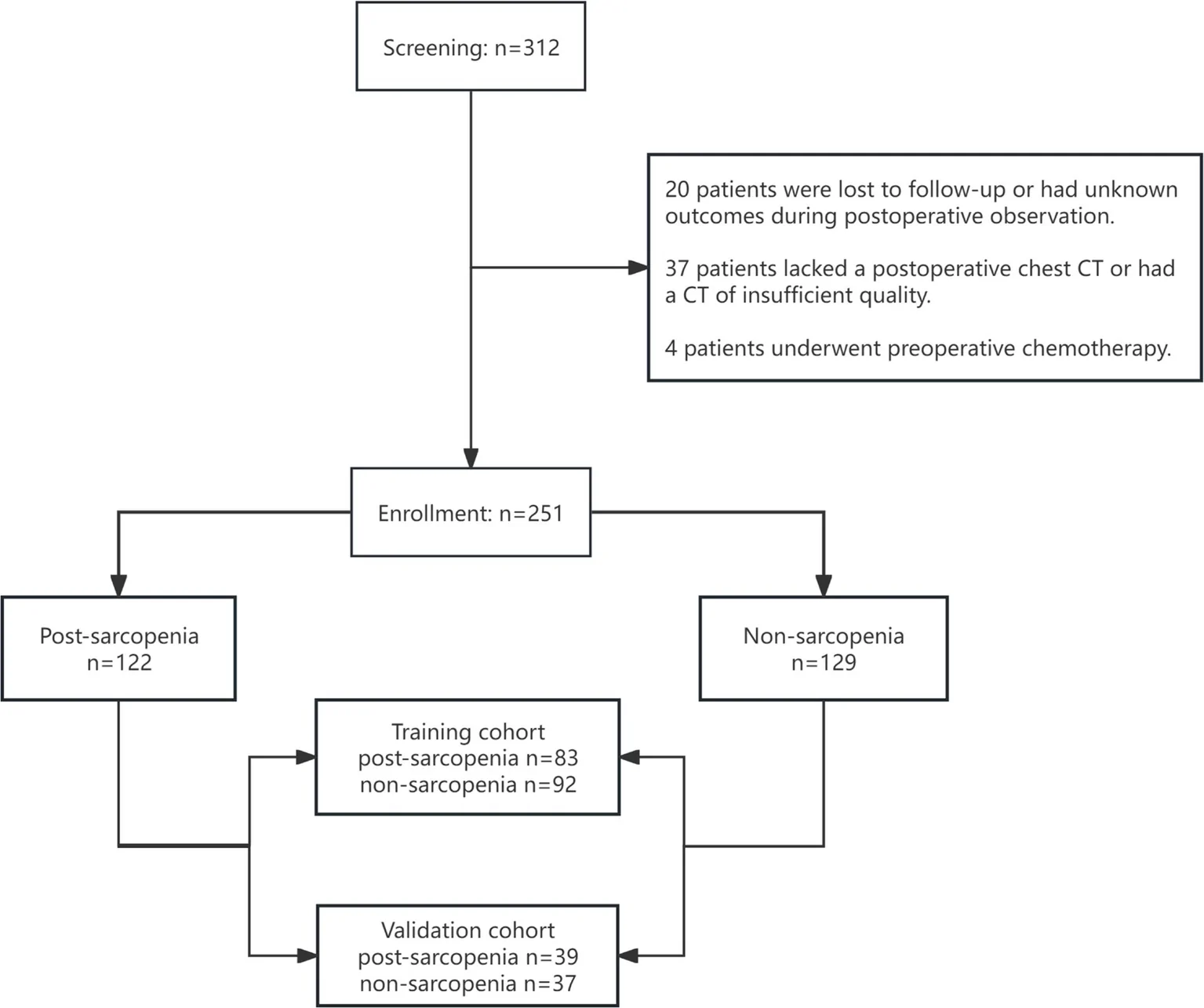
Flowchart of the study.

This retrospective study involved patients with early-stage NSCLC who underwent surgical treatment at our institution. The study population included hospitalized patients aged 18 and older with stage I-II NSCLC who underwent video-assisted thoracic surgery (lobectomy or sublobectomy, including segmentectomy and wedge resection) between January 2018 and December 2020, and underwent CT at 1 year post-surgery. All surgeries were performed by our experienced surgical team following standard procedures. Patients were excluded if they: (1) were lost to follow-up or had unknown outcomes during postoperative observation; (2) lacked a postoperative chest CT or had a CT of insufficient quality; (3) underwent preoperative chemotherapy. A study flow chart is illustrated in Figure 1. To evaluate potential selection or survivorship bias introduced by this inclusion process, baseline characteristics were compared between included and excluded patients. Regarding missing data, we performed a complete-case analysis. The diagnostic criteria for post-sarcopenia were an SMI of <42.6 cm^2^/m^2^ for men and <30.6 cm^2^/m^2^ for women (11).

### Data collection and imaging measurements

The data for candidate predictors in both the training and validation sets encompassed demographic information, the American Society of Anesthesiologists (ASA) score (12), comorbidities, nutritional status, oncology, perioperative inflammation indicators, surgical procedures, blood loss, postoperative hospital stay, length of drainage, and complications within 30 days after surgery. These data were extracted from preoperative medical records and patient follow-up. Candidate predictors for sarcopenia were selected based on a comprehensive review of related literature (7, 13). Demographic data included age, sex, and smoking history. Comorbidities included hypertension, diabetes, coronary artery disease, and the Charlson Comorbidity Index (CCI). Nutritional data comprised the body mass index (BMI), calculated as weight in kilograms divided by height in meters squared, and the SMI, determined by dividing SMA by height in meters squared. SMA was evaluated by analyzing the area of the CT scan images of the 12th thoracic vertebra one year after surgery, utilizing a Hounsfield unit range of −29 to +150. Oncology data encompassed histology and cancer staging according to the 8th edition of the AJCC classification. Perioperative inflammation indicators included preoperative white blood cell (WBC) counts, neutrophil count (NEU), lymphocyte count (LY), mean corpuscular volume (MCV), platelet (PLT), C-reactive protein (CRP), and albumin (ALB). Additional preoperative indicators encompassed serum creatinine, serum cystatin C, blood urea nitrogen (BUN), hemoglobin (HGB), and the serum creatinine/cystatin C ratio (CCR), as well as postoperative WBC, NEU, LY, CRP, MCV, PLT, ALB, and prealbumin (PA). Postoperative complications included fever, subcutaneous emphysema, prolonged air leak (PAL, defined as persistent air leakage continuing beyond 5 days postoperatively) (14), pneumonia, and complications graded according to the Clavien-Dindo grading system.

### Statistical analysis

Data sorting and statistical analysis were performed using the Storm Statistics Platform^1^ and R version 4.3.3. Continuous variables were described using mean ± standard deviation or median (interquartile range). Categorical variables were presented as frequencies and percentages. Continuous variables were analyzed using either the Student’s *t*-test or the Wilcoxon rank-sum test. Categorical variables were analyzed using the Chi-square test. A *p*-value less than 0.05 was deemed statistically significant.

### Logistic regression analysis and nomogram model construction and evaluation

Patients were randomly allocated into training and validation sets at a 7:3 ratio for logistic regression analysis and subsequent development and assessment of the nomogram. Candidate predictors were initially screened using univariable analysis to identify variables associated with the outcome. All selected predictors were further evaluated by clinical plausibility and subject-matter expertise prior to final model building. To mitigate overfitting and to assess robustness, we performed internal validation using 1,000 bootstrap resamples to estimate optimism-corrected performance metrics (calibration slope/intercept and Brier score). Univariate logistic regression was employed on the training set to identify suitable variables for inclusion in the multivariate logistic regression model. After multivariate analysis, independent predictors were selected, and a nomogram was constructed using the “rms” software package.

## Result

### Baseline characteristics of patients

As shown in Table 1, this study collected data from 251 patients with NSCLC who underwent treatment at our hospital between 2018 and 2020. Out of the total patients, 70% (*n* = 175) were randomly allocated to a training set and 30% (*n* = 76) were included in a validation set. Overall, the study population exhibited a mean age of 64.4 years, with a female predominance (63.75%, *n* = 160). In the training set, the median age was 62 years, with 66.29% (*n* = 116) being female. A total of 122 patients developed postoperative sarcopenia (*n* = 83 in the training set, *n* = 39 in the validation set). The detailed characteristics of the two cohorts are shown in Table 2. The baseline characteristics and statistical comparisons of the external test cohort is shown in Table 3.

**Table 1:** Patient characteristics.

Variables	Overall (*n* = 251)	Non-sarcopenia (*n* = 129)	Sarcopenia (*n* = 122)	*p*
General characteristics				
Weight^a^	67.63 ± 10.83	70.17 ± 10.82	64.93 ± 10.21	<0.001
BMI^a^	24.84 ± 3.33	26.39 ± 3.2	23.21 ± 2.63	<0.001
Age^a^	60.44 ± 9.93	60.94 ± 9.04	59.91 ± 10.79	0.415
Gender (N, %)				<0.001
Female	160 (64)	104 (81)	56 (46)	
Male	91 (36)	25 (19)	66 (54)	
ASA (N, %)				0.249
1	12 (5)	4 (3)	8 (7)	
2	211 (84)	113 (88)	98 (80)	
3	28 (11)	12 (9)	16 (13)	
Coronary disease (N, %)				1
NO	209 (83)	107 (83)	102 (84)	
YES	42 (17)	22 (17)	20 (16)	
Hypertension (N, %)				0.86
NO	156 (62)	79 (61)	77 (63)	
YES	95 (38)	50 (39)	45 (37)	
Diabetes mellitus (N, %)				0.868
NO	220 (88)	114 (88)	106 (87)	
YES	31 (12)	15 (12)	16 (13)	
CCI>1 (N, %)				0.497
NO	130 (52)	70 (54)	60 (49)	
YES	121 (48)	59 (46)	62 (51)	
Smoke history, N (%)				<0.001
NO	192 (76)	113 (88)	79 (65)	
YES	59 (24)	16 (12)	43 (35)	
Stage (N, %)				0.46
I	49 (20)	28 (22)	21 (17)	
II	202 (80)	101 (78)	101 (83)	
Pathological type, N (%)				0.161
Squamous cell carcinoma	17 (7)	5 (4)	12 (10)	
Adenocarcinoma	229 (91)	121 (94)	108 (89)	
Others	5 (2)	3 (2)	2 (2)	
Surgical procedure (N, %)				0.633
Subsegmentectomy	96 (38)	47 (36)	49 (40)	
Lobectomy	155 (62)	82 (64)	73 (60)	
Surgical complication				
Blood loss^b^ (ml)	20 (10, 50)	20 (10, 50)	20 (10, 27.5)	0.048
Postoperative length of hospital stay (day)^b^	4 (3, 6)	4 (3, 6)	4 (3, 7)	0.854
Length of drainage (day)^b^	3 (2, 4)	3 (2, 4)	3 (2, 5)	0.393
Fever (N, %)				0.473
NO	200 (80)	100 (78)	100 (82)	
YES	51 (20)	29 (22)	22 (18)	
Subcutaneous emphysema (N, %)				1
NO	240 (96)	123 (95)	117 (96)	
YES	11 (4)	6 (5)	5 (4)	
PAL (N, %)				0.195
NO	231 (92)	122 (95)	109 (89)	
YES	20 (8)	7 (5)	13 (11)	
Pneumonia (N, %)				0.684
NO	245 (98)	125 (97)	120 (98)	
YES	6 (2)	4 (3)	2 (2)	
NRS (N, %)				0.085
0	25 (10)	13 (10)	12 (10)	
1	23 (9)	7 (5)	16 (13)	
2	126 (50)	72 (56)	54 (44)	
3	35 (14)	16 (12)	19 (16)	
4	22 (9)	9 (7)	13 (11)	
5	9 (4)	4 (3)	5 (4)	
6	5 (2)	3 (2)	2 (2)	
7	5 (2)	5 (4)	0 (0)	
8	1 (0)	0 (0)	1 (1)	
Clavien–Dindo ≥2 (N, %)				0.546
NO	232 (92)	121 (94)	111 (91)	
YES	19 (8)	8 (6)	11 (9)	
Perioperative laboratory indicators				
Postoperative WBC (×10^9^/L)^b^	10.8 (8.62, 12.75)	10.81 (9.21, 12.77)	10.64 (8.26, 12.69)	0.409
Postoperative Neut (×10^9^/L)^b^	8.48 (6.46, 10.56)	8.59 (7.12, 10.52)	8.3 (6.24, 10.54)	0.318
Postoperative Ly (×10^9^/L)^b^	1.27 (1.03, 1.61)	1.27 (1.06, 1.61)	1.26 (0.98, 1.6)	0.568
Postoperative CRP (mg/L)^b^	26.59 (16.15, 43.12)	28.13 (17.76, 43.56)	24.98 (14.73, 40.65)	0.375
Postoperative MCV (fL)^b^	92.4 (89.7, 95.3)	92.6 (89.7, 94.9)	92.3 (89.75, 95.38)	0.815
Postoperative PLT (×10^9^/L)^b^	210 (178, 253)	213 (185, 254)	204.5 (176, 248.5)	0.336
Postoperative ALB (g/L)^b^	35.81 (33.76, 38.08)	36.32 (34.16, 38.7)	35.49 (33.63, 37.71)	0.089
Postoperative PA (mg/L)^b^	221 (197.5, 261.5)	228 (202, 259)	215 (191, 261.75)	0.243
WBC (×10^9^/L)^b^	5.95 (4.85, 6.95)	5.89 (4.94, 6.7)	6.14 (4.74, 7.29)	0.401
Neut (×10^9^/L)^b^	3.16 (2.43, 4)	3.02 (2.43, 3.78)	3.26 (2.42, 4.45)	0.096
Ly (×10^9^/L)^b^	1.93 (1.53, 2.36)	2.02 (1.67, 2.36)	1.73 (1.4, 2.36)	0.012
HGB (g/L)^b^	134 (126, 143)	132 (125, 140)	136 (126.25, 146)	0.023
MCV (fL)^b^	91.5 (89.8, 94.2)	91.5 (89.6, 94.3)	91.55 (89.9, 94.18)	0.55
PLT (×10^9^/L)^b^	223 (188, 268.5)	234 (199, 271)	216 (180.25, 266.75)	0.107
CRP (mg/L)^b^	0.9 (0.5, 3.14)	0.78 (0.5, 2.33)	1 (0.5, 5.18)	0.056
ALB (g/L)^a^	40.26 ± 3.41	40.19 ± 3.22	40.34 ± 3.61	0.725
Serum creatinine (mg/dl)^b^	0.72 (0.63, 0.81)	0.7 (0.63, 0.77)	0.74 (0.64, 0.87)	0.041
Serum cystatin C (mg/L)^b^	0.75 (0.66, 0.87)	0.77 (0.67, 0.9)	0.74 (0.64, 0.83)	0.228
BUN (mmol/L)^b^	5.38 (4.62, 6.2)	5.42 (4.74, 6.3)	5.36 (4.5, 6.04)	0.283
CCR^b^	0.94 (0.81, 1.12)	0.88 (0.77, 1.07)	1 (0.85, 1.15)	0.005

a is expressed as mean ± standard deviation; b values are expressed as interquartile spacing (median [¼,¾ digits]). BMI, body mass index; ASA, the American Society of Anesthesiologists score; CCI, Charlson Comorbidity Index; PAL, prolonged air leak; NRS, numerical rating scale; WBC, white blood cell counts; Neut, neutrophil count; Ly, lymphocyte count; MCV, mean corpuscular volume; PLT, platelet; CRP, C-reactive protein; ALB, albumin; HGB, hemoglobin; PA, prealbumin; BUN, blood urea nitrogen; CCR, serum creatinine/cystatin C ratio.

**Table 2:** The baseline of training cohort and validation cohort.

Variables	Total (*n* = 251)	Training (*n* = 175)	Validation (*n* = 76)	*p*
General characteristics				
Weight^a^	67.63 ± 10.83	67.65 ± 10.42	67.56 ± 11.79	0.949
BMI^a^	24.84 ± 3.33	24.90 ± 3.19	24.70 ± 3.66	0.664
Age^a^	60.44 ± 9.93	60.63 ± 9.81	59.99 ± 10.24	0.636
Gender (N, %)				0.204
Female	160 (63.75)	116 (66.29)	44 (57.89)	
Male	91 (36.25)	59 (33.71)	32 (42.11)	
ASA (N, %)				0.672
1	12 (4.78)	7 (4.00)	5 (6.58)	
2	211 (84.06)	148 (84.57)	63 (82.89)	
3	28 (11.16)	20 (11.43)	8 (10.53)	
Coronary disease (N, %)				0.317
NO	209 (83.27)	143 (81.71)	66 (86.84)	
YES	42 (16.73)	32 (18.29)	10 (13.16)	
Hypertension (N, %)				0.726
NO	156 (62.15)	110 (62.86)	46 (60.53)	
YES	95 (37.85)	65 (37.14)	30 (39.47)	
Diabetes mellitus (N, %)				0.500
NO	220 (87.65)	155 (88.57)	65 (85.53)	
YES	31 (12.35)	20 (11.43)	11 (14.47)	
CCI (N, %)				0.921
NO	130 (51.79)	91 (52.00)	39 (51.32)	
YES	121 (48.21)	84 (48.00)	37 (48.68)	
Smoking history (N, %)				0.310
NO	192 (76.49)	137 (78.29)	55 (72.37)	
YES	59 (23.51)	38 (21.71)	21 (27.63)	
Stage (N, %)				0.184
I	49 (19.52)	38 (21.71)	11 (14.47)	
II	202 (80.48)	137 (78.29)	65 (85.53)	
Pathological type, N (%)				0.923
Squamous cell carcinoma	17 (6.77)	11 (6.29)	6 (7.89)	
Adenocarcinoma	229 (91.24)	160 (91.43)	69 (90.79)	
Others	5 (1.99)	4 (2.29)	1 (1.32)	
Surgical procedure (N, %)				0.250
Subsegmentectomy	96 (38.25)	71 (40.57)	25 (32.89)	
Lobectomy	155 (61.75)	104 (59.43)	51 (67.11)	
Post-sarcopenia (N, %)				0.571
NO	129 (51.39)	92 (52.57)	37 (48.68)	
YES	122 (48.61)	83 (47.43)	39 (51.32)	
Surgical complication				
Blood loss^b^ (ml)	20.00 (10.00, 50.00)	20.00 (10.00, 50.00)	20.00 (17.50, 50.00)	0.198
Postoperative length of hospital stay (day)^b^	4.00 (3.00, 6.00)	4.00 (3.00, 6.50)	4.00 (3.00, 6.00)	0.918
Length of drainage (day)^b^	3.00 (2.00, 4.00)	3.00 (2.00, 4.50)	3.00 (2.00, 4.00)	0.892
Fever (N, %)				0.849
0	200 (79.68)	140 (80.00)	60 (78.95)	
1	51 (20.32)	35 (20.00)	16 (21.05)	
Subcutaneous emphysema (N, %)				0.910
0	240 (95.62)	168 (96.00)	72 (94.74)	
1	11 (4.38)	7 (4.00)	4 (5.26)	
PAL (N, %)				0.297
0	231 (92.03)	159 (90.86)	72 (94.74)	
1	20 (7.97)	16 (9.14)	4 (5.26)	
Pneumonia (N, %)				0.776
0	245 (97.61)	170 (97.14)	75 (98.68)	
1	6 (2.39)	5 (2.86)	1 (1.32)	
NRS (N, %)				0.978
0	25 (9.96)	18 (10.29)	7 (9.21)	
1	23 (9.16)	17 (9.71)	6 (7.89)	
2	126 (50.20)	84 (48.00)	42 (55.26)	
3	35 (13.94)	26 (14.86)	9 (11.84)	
4	22 (8.76)	14 (8.00)	8 (10.53)	
5	9 (3.59)	7 (4.00)	2 (2.63)	
6	5 (1.99)	4 (2.29)	1 (1.32)	
7	5 (1.99)	4 (2.29)	1 (1.32)	
8	1 (0.40)	1 (0.57)	0 (0.00)	
Surgical complication grade >I (N, %)				0.898
NO	232 (92.43)	162 (92.57)	70 (92.11)	
YES	19 (7.57)	13 (7.43)	6 (7.89)	
Perioperative laboratory indicators				
Postoperative WBC (×10^9^/L)^b^	10.80 (8.62, 12.75)	10.87 (8.50, 12.75)	10.57 (8.89, 12.61)	0.896
Postoperative NEUT (×10^9^/L)^b^	8.48 (6.46, 10.56)	8.54 (6.45, 10.56)	8.29 (6.46, 10.54)	0.975
Postoperative LY (×10^9^/L)^b^	1.27 (1.03, 1.61)	1.27 (1.03, 1.62)	1.27 (1.05, 1.54)	0.852
Postoperative CRP (mg/L)^b^	26.59 (16.15, 43.12)	25.06 (14.69, 40.67)	30.73 (17.73, 53.06)	0.082
Postoperative MCV (fL)^b^	92.40 (89.70, 95.30)	93.00 (89.65, 95.30)	92.10 (90.15, 94.90)	0.604
Postoperative PLT (×10^9^/L)^b^	210.00 (178.00, 253.00)	213.00 (178.00, 257.00)	206.00 (177.50, 247.50)	0.505
Postoperative ALB (g/L)^b^	35.81 (33.77, 38.08)	35.84 (33.83, 38.24)	35.78 (33.77, 37.82)	0.899
Postoperative PA (mg/L)^b^	221.00 (197.50, 261.50)	218.00 (198.00, 259.00)	229.00 (191.75, 268.75)	0.563
WBC (×10^9^/L)^b^	5.95 (4.85, 6.95)	5.91 (4.91, 7.03)	5.97 (4.72, 6.94)	0.831
NEU T (×10^9^/L)^b^	3.16 (2.43, 4.00)	3.19 (2.44, 3.92)	3.16 (2.42, 4.23)	0.736
LY (×10^9^/L)^b^	1.93 (1.53, 2.36)	1.96 (1.54, 2.33)	1.89 (1.47, 2.37)	0.849
HGB (g/L)^b^	134.00 (126.00, 143.00)	133.00 (126.00, 142.50)	135.50 (126.00, 146.50)	0.398
MCV (fL)^b^	91.50 (89.80, 94.20)	91.70 (89.50, 94.30)	91.15 (89.90, 93.80)	0.644
PLT (×10^9^/L)^b^	223.00 (188.00, 268.50)	225.00 (190.50, 268.50)	221.00 (182.75, 266.75)	0.823
CRP (mg/L)^b^	0.90 (0.50, 3.15)	0.85 (0.50, 3.60)	1.08 (0.50, 2.69)	0.934
ALB (g/L)^a^	40.12 (37.81, 42.77)	40.16 (37.74, 42.54)	39.91 (37.94, 43.43)	0.613
Serum creatinine (mg/dl)^b^	0.72 (0.63, 0.81)	0.71 (0.63, 0.81)	0.72 (0.64, 0.81)	0.987
Serum cystatin C (mg/L)^b^	0.75 (0.67, 0.87)	0.75 (0.67, 0.90)	0.72 (0.64, 0.82)	0.139
BUN (mmol/L)^b^	5.38 (4.62, 6.20)	5.41 (4.74, 6.26)	5.31 (4.28, 6.10)	0.425
CCR^b^	0.94 (0.81, 1.12)	0.91 (0.80, 1.11)	0.99 (0.85, 1.13)	0.170

a is expressed as mean ± standard deviation; b values are expressed as interquartile spacing (median [¼,¾ digits]). BMI, body mass index; ASA, the American Society of Anesthesiologists score; CCI, Charlson Comorbidity Index; PAL, prolonged air leak; NRS, numerical rating scale; PA, Prealbumin; BUN, Blood Urea Nitrogen; CCR, serum creatinine/cystatin C ratio.

**Table 3:** The baseline characteristics of the testing cohort.

Variables	Overall (*n* = 76)	Non-sarcopenia (*n* = 37)	Sarcopenia (*n* = 39)	*p*
General characteristics				
Weight^a^	67.56 ± 11.79	72.31 ± 10.71	63.05 ± 11.09	<0.001
BMI^a^	24.70 ± 3.66	26.82 ± 3.07	22.69 ± 2.99	<0.001
Age^a^	59.99 ± 10.24	60.27 ± 10.16	59.72 ± 10.44	0.816
Gender (N, %)				0.010
Female	44 (57.89)	27 (72.97)	17 (43.59)	
Male	32 (42.11)	10 (27.03)	22 (56.41)	
ASA (N, %)				0.904
1	5 (6.58)	3 (8.11)	2 (5.13)	
2	63 (82.89)	30 (81.08)	33 (84.62)	
3	8 (10.53)	4 (10.81)	4 (10.26)	
Coronary disease (N, %)				1.000
NO	66 (86.84)	32 (86.49)	34 (87.18)	
YES	10 (13.16)	5 (13.51)	5 (12.82)	
Hypertension (N, %)				0.513
NO	46 (60.53)	21 (56.76)	25 (64.10)	
YES	30 (39.47)	16 (43.24)	14 (35.90)	
Diabetes mellitus (N, %)				0.377
NO	65 (85.53)	33 (89.19)	32 (82.05)	
YES	11 (14.47)	4 (10.81)	7 (17.95)	
CCI > 1 (N, %)				0.642
NO	39 (51.32)	20 (54.05)	19 (48.72)	
YES	37 (48.68)	17 (45.95)	20 (51.28)	
Smoking history (N, %)				0.254
NO	55 (72.37)	29 (78.38)	26 (66.67)	
YES	21 (27.63)	8 (21.62)	13 (33.33)	
Stage (N, %)				0.674
I	11 (14.47)	6 (16.22)	5 (12.82)	
II	65 (85.53)	31 (83.78)	34 (87.18)	
Pathological type, N (%)				0.675
Squamous cell carcinoma	6 (7.89)	2 (5.41)	4 (10.26)	
Adenocarcinoma	69 (90.79)	35 (94.59)	34 (87.18)	
Others	1 (1.32)	0 (0.00)	1 (2.56)	
Surgical procedure (N, %)				0.933
Subsegmentectomy	25 (32.89)	12 (32.43)	13 (33.33)	
Lobectomy	51 (67.11)	25 (67.57)	26 (66.67)	
Surgical complication				
Blood loss^b^ (ml)	20 (17.5, 50)	20 (20, 50)	20 (10, 30)	0.206
Postoperative length of hospital stay (day)^b^	4.00 (3.00, 6.00)	4.00 (3.00, 5.00)	4.00 (3.00, 6.50)	0.646
Length of drainage (day)^b^	3.00 (2.00, 4.00)	3.00 (2.00, 4.00)	3.00 (2.00, 4.00)	0.864
Fever (N, %)				0.116
NO	60 (78.95)	32 (86.49)	28 (71.79)	
YES	16 (21.05)	5 (13.51)	11 (28.21)	
Subcutaneous emphysema (N, %)				0.570
NO	72 (94.74)	34 (91.89)	38 (97.44)	
YES	4 (5.26)	3 (8.11)	1 (2.56)	
PAL (N, %)				0.646
NO	72 (94.74)	36 (97.30)	36 (92.31)	
YES	4 (5.26)	1 (2.70)	3 (7.69)	
Pneumonia (N, %)				0.487
NO	75 (98.68)	36 (97.30)	39 (100.00)	
YES	1 (1.32)	1 (2.70)	0 (0.00)	
NRS (N, %)				0.424
0	7 (9.21)	4 (10.81)	3 (7.69)	
1	6 (7.89)	1 (2.70)	5 (12.82)	
2	42 (55.26)	22 (59.46)	20 (51.28)	
3	9 (11.84)	5 (13.51)	4 (10.26)	
4	8 (10.53)	3 (8.11)	5 (12.82)	
5	2 (2.63)	0 (0.00)	2 (5.13)	
6	1 (1.32)	1 (2.70)	0 (0.00)	
7	1 (1.32)	1 (2.70)	0 (0.00)	
Surgical complication grade >I (N, %)				0.622
NO	70 (92.11)	33 (89.19)	37 (94.87)	
YES	6 (7.89)	4 (10.81)	2 (5.13)	
Perioperative laboratory indicators				
Postoperative WBC (×10^9^/L)^b^	10.57 (8.89, 12.61)	10.67 (9.21, 12.95)	9.95 (8.79, 12.26)	0.433
Postoperative NEUT (×10^9^/L)^b^	8.29 (6.46, 10.54)	8.74 (6.84, 11.43)	7.88 (6.41, 10.18)	0.316
Postoperative LY (×10^9^/L)^b^	1.27 (1.05, 1.54)	1.22 (1.05, 1.38)	1.32 (1.04, 1.92)	0.262
Postoperative CRP (mg/L)^b^	30.73 (17.73, 53.06)	29.40 (16.69, 43.56)	33.08 (18.77, 58.11)	0.321
Postoperative MCV (fL)^b^	92.10 (90.15, 94.90)	92.00 (90.20, 94.30)	92.10 (90.15, 95.00)	0.946
Postoperative PLT (×10^9^/L)^b^	206.00 (177.50, 247.50)	204.00 (186.00, 230.00)	208.00 (173.50, 258.50)	0.930
Postoperative ALB (g/L)^b^	35.78 (33.77, 37.82)	36.09 (34.66, 37.95)	35.31 (33.44, 37.42)	0.192
Postoperative PA (mg/L)^b^	229.00 (191.75, 268.75)	243.00 (205.00, 284.00)	214.00 (187.00, 258.00)	0.141
WBC (×10^9^/L)^b^	5.97 (4.72, 6.94)	5.42 (4.67, 6.25)	6.23 (4.75, 7.80)	0.187
NEUT (×10^9^/L)^b^	3.16 (2.42, 4.23)	2.89 (2.32, 3.49)	3.34 (2.79, 5.05)	0.037
LY (×10^9^/L)^b^	1.89 (1.47, 2.37)	1.99 (1.74, 2.44)	1.74 (1.31, 2.20)	0.098
HGB (g/L)^b^	135.50 (126.00, 146.50)	134.00 (127.00, 138.00)	138.00 (124.50, 148.00)	0.483
MCV (fL)^b^	91.15 (89.90, 93.80)	90.80 (89.00, 93.80)	91.30 (90.50, 93.80)	0.228
PLT (×10^9^/L)^b^	221.00 (182.75, 266.75)	227.00 (186.00, 245.00)	215.00 (182.50, 280.00)	0.716
CRP (mg/L)^b^	1.08 (0.50, 2.69)	1.24 (0.50, 2.31)	0.95 (0.50, 3.15)	0.774
ALB (g/L)^a^	39.91 (37.94, 43.43)	39.62 (37.82, 43.00)	40.37 (38.17, 43.50)	0.613
Serum creatinine (mg/dl)^b^	0.72 (0.64, 0.81)	0.71 (0.63, 0.76)	0.73 (0.64, 0.86)	0.306
Serum cystatin C (mg/L)^b^	0.72 (0.64, 0.82)	0.75 (0.67, 0.84)	0.72 (0.64, 0.81)	0.470
BUN (mmol/L)^b^	5.31 (4.28, 6.10)	5.18 (4.30, 5.99)	5.37 (4.23, 6.38)	0.564
CCR^b^	0.99 (0.85, 1.13)	0.94 (0.77, 1.12)	1.02 (0.89, 1.12)	0.341

a is expressed as mean ± standard deviation; b values are expressed as interquartile spacing (median [¼,¾ digits]). BMI, body mass index; ASA, the American Society of Anesthesiologists score; CCI, Charlson Comorbidity Index; PAL, prolonged air leak; NRS, numerical rating scale; PA, Prealbumin; BUN, Blood Urea Nitrogen; CCR, serum creatinine/cystatin C ratio.

### Identification of predictive factors

Univariate and multivariate analyses in the training set identified six factors that were significantly associated with post-sarcopenia (*p* < 0.05), and the full set of univariable results were presented in the Table 4. In early-stage NSCLC patients, independent predictive factors for post-sarcopenia include male sex (OR = 8.46, 95% CI: 2.03–35.21, *p* = 0.003), BMI (OR = 0.49, 95% CI: 0.34–0.72, *p* < 0.001), lobectomy (OR = 0.25, 95% CI: 0.09–0.67, *p* = 0.006), PAL (OR = 8.17, 95% CI: 1.02–65.69, *p* = 0.048), postoperative albumin (OR = 0.86, 95% CI: 0.77–0.97, *p* = 0.013), and CRP (OR = 1.07, 95% CI: 1.01–1.13, *p* = 0.015). Table 5 presents the multivariate analysis results for post-sarcopenia in the training set.

**Table 4:** Univariate logistic regression analysis of post-sarcopenia.

Variables	*β*	S.E	*z*	*p*	OR (95%CI)
Gender (Male)	1.76	0.36	4.91	<0.001	5.79 (2.87–11.68)
ASA					
1					1.00 (Reference)
2	−2.04	1.09	−1.86	0.062	0.13 (0.02–1.11)
3	−1.39	1.17	−1.18	0.237	0.25 (0.03–2.49)
Coronary disease (Yes)	−0.03	0.39	−0.07	0.945	0.97 (0.45–2.10)
Hypertension (Yes)	0.02	0.31	0.05	0.957	1.02 (0.55–1.88)
Diabetes mellitus (Yes)	−0.11	0.48	−0.23	0.817	0.90 (0.35–2.28)
CCI > 1 (Yes)	0.20	0.30	0.65	0.513	1.22 (0.67–2.21)
Smoke history (Yes)	1.78	0.43	4.10	<0.001	5.94 (2.53–13.94)
Pathological type					
Squamous cell carcinoma					1.00 (Reference)
Adenocarcinoma	−1.13	0.70	−1.63	0.104	0.32 (0.08–1.26)
Others	−2.08	1.34	−1.55	0.120	0.13 (0.01–1.72)
Stage (II)	0.27	0.37	0.74	0.458	1.32 (0.64–2.72)
Surgical procedure (Subsegmentectomy)	−0.22	0.31	−0.72	0.474	0.80 (0.44–1.47)
Fever (Yes)	−0.84	0.40	−2.09	0.037	0.43 (0.20–0.95)
Subcutaneous emphysema (Yes)	0.41	0.78	0.52	0.602	1.50 (0.33–6.92)
PAL (Yes)	0.67	0.54	1.25	0.212	1.96 (0.68–5.66)
Pneumonia (Yes)	−0.31	0.93	−0.34	0.737	0.73 (0.12–4.49)
Clavien–Dindo ≥2 (Yes)	0.98	0.62	1.58	0.113	2.68 (0.79–9.04)
Blood loss	−0.00	0.00	−1.43	0.152	1.00 (0.99–1.00)
Postoperative length of hospital stay	0.02	0.04	0.48	0.629	1.02 (0.94–1.10)
Length of drainage	0.04	0.04	0.96	0.336	1.04 (0.96–1.14)
NRS	−0.05	0.10	−0.53	0.597	0.95 (0.78–1.15)
Weight	−0.03	0.02	−2.19	0.029	0.97 (0.94–0.99)
BMI	−0.37	0.07	−5.22	<0.001	0.69 (0.60–0.79)
Age	−0.01	0.02	−0.81	0.416	0.99 (0.96–1.02)
Postoperative WBC	−0.01	0.05	−0.22	0.827	0.99 (0.90–1.09)
Postoperative Neut	−0.00	0.05	−0.01	0.995	1.00 (0.91–1.10)
Postoperative Ly	0.00	0.14	0.03	0.978	1.00 (0.76–1.32)
Postoperative CRP	−0.01	0.01	−0.86	0.392	0.99 (0.98–1.01)
Postoperative MCV	0.01	0.03	0.49	0.625	1.01 (0.96–1.07)
Postoperative PLT	−0.00	0.00	−0.90	0.368	1.00 (0.99–1.00)
Postoperative ALB	−0.01	0.04	−0.40	0.693	0.99 (0.92–1.06)
Postoperative PA	−0.00	0.00	−0.29	0.768	1.00 (0.99–1.01)
WBC	−0.00	0.07	−0.03	0.972	1.00 (0.87–1.14)
Neut	0.03	0.08	0.43	0.665	1.03 (0.89–1.21)
Ly	−0.46	0.25	−1.84	0.065	0.63 (0.39–1.03)
HGB	0.03	0.01	2.34	0.019	1.03 (1.01–1.05)
MCV	0.00	0.03	0.14	0.892	1.00 (0.95–1.06)
PLT	−0.01	0.00	−2.06	0.039	0.99 (0.99–0.99)
CRP	0.05	0.02	2.16	0.031	1.05 (1.01–1.09)
ALB	0.01	0.05	0.15	0.878	1.01 (0.92–1.10)
Serum creatinine	2.30	1.09	2.11	0.035	9.94 (1.18–84.12)
Serum cystatin C	0.24	0.47	0.52	0.604	1.28 (0.51–3.21)
BUN	−0.21	0.12	−1.74	0.083	0.81 (0.64–1.03)
CCR	0.62	0.53	1.17	0.242	1.86 (0.66–5.26)

OR: Odds Ratio, CI: Confidence Interval. BMI, body mass index; ASA, the American Society of Anesthesiologists score; CCI, Charlson Comorbidity Index; PAL, prolonged air leak; NRS, numerical rating scale; WBC, white blood cell counts; Neut, neutrophil count; Ly, lymphocyte count; MCV, mean corpuscular volume; PLT, platelet; CRP, C-reactive protein; ALB, albumin; HGB, hemoglobin; PA, prealbumin; BUN, blood urea nitrogen; CCR, serum creatinine/cystatin C ratio.

**Table 5:** Multivariate logistic regression analysis of risk factors associated with post-sarcopenia.

Variables	*β*	Odds ratio (95%CI)	*p* value
Intercept	13.42		0.006
Gender	2.14	8.46 (2.03–35.21)	0.003
Surgical procedure	−1.40	0.25 (0.09–0.67)	0.006
PAL	2.10	8.17 (1.02–65.69)	0.048
BMI	−0.71	0.49 (0.34–0.72)	<0.001
Postoperative ALB	−0.15	0.86 (0.77–0.97)	0.013
CRP	0.07	1.07 (1.01–1.13)	0.015

BMI, body mass index; PAL, prolonged air leak; ALB, albumin; CRP, C-reactive protein.

### Construction of the prediction model

**Figure 2:**
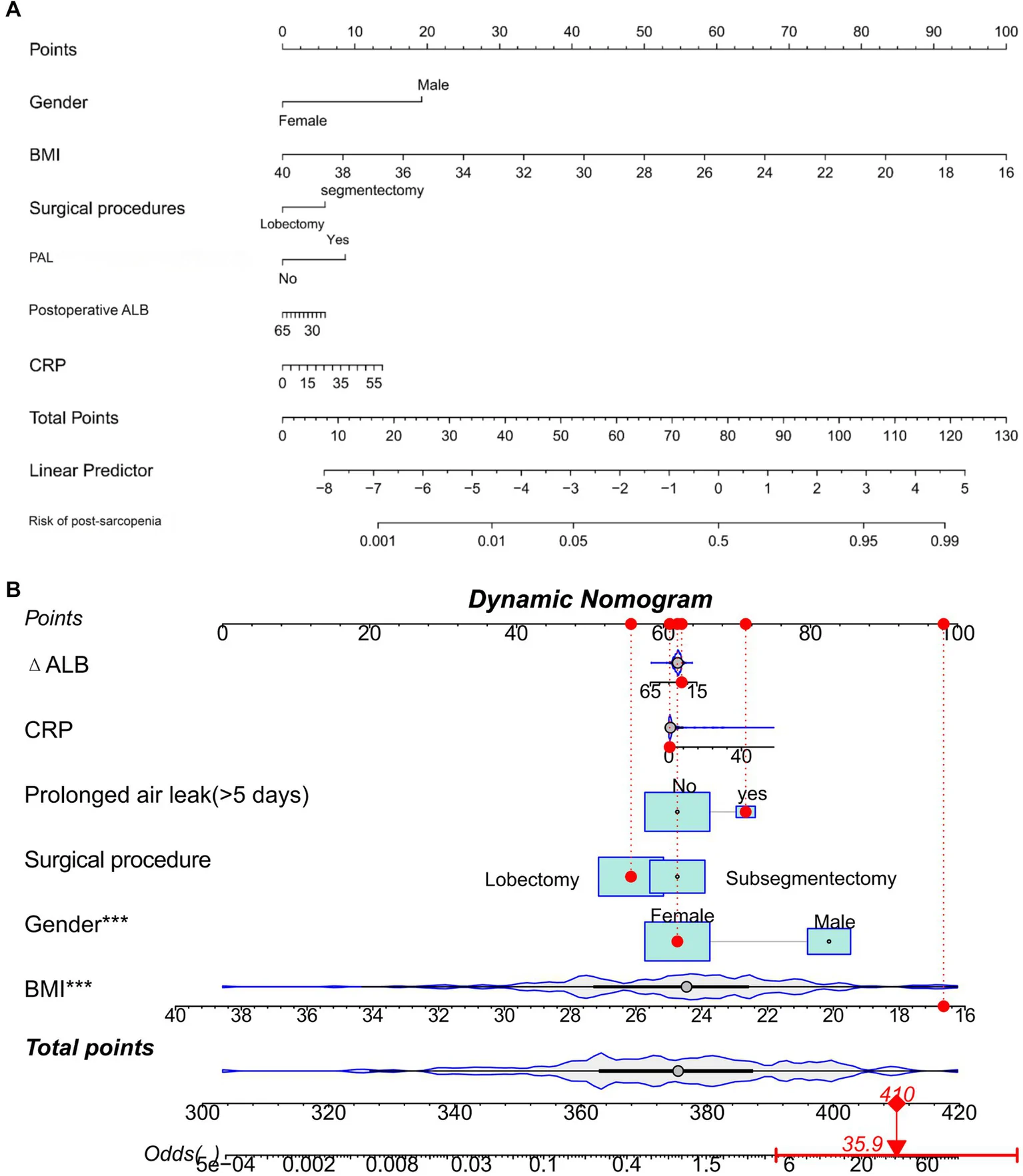
**(A)** Nomogram prediction model for post-sarcopenia. **(B)** Example of a dynamic nomogram, revealing a 97.3% probability of post-sarcopenia in an early-stage non-small cell lung cancer patient.

A nomogram was established by incorporating the above key clinical features (Figure 2A). Model stability was evaluated through events per variable (EPV) analysis. In the training cohort (*n* = 175) where 83 patients developed postoperative sarcopenia, our six-predictor model achieved an EPV of 13.8 (83/6), substantially exceeding the recommended minimum threshold of 10 EPV. This suggests adequate statistical power with a low risk of overfitting. The nomogram illustrated the relative impact of each factor on the risk of post-sarcopenia. According to the nomogram, BMI was identified as the strongest predictor for post-sarcopenia. The nomogram also emphasized protective factors, including female sex, lobectomy, and PAL. The values for each variable were summed to yield a total score, and the corresponding predicted probability was displayed at the bottom (Figure 2B).

### Validation of the prediction model

#### ROC curve

**Figure 3:**
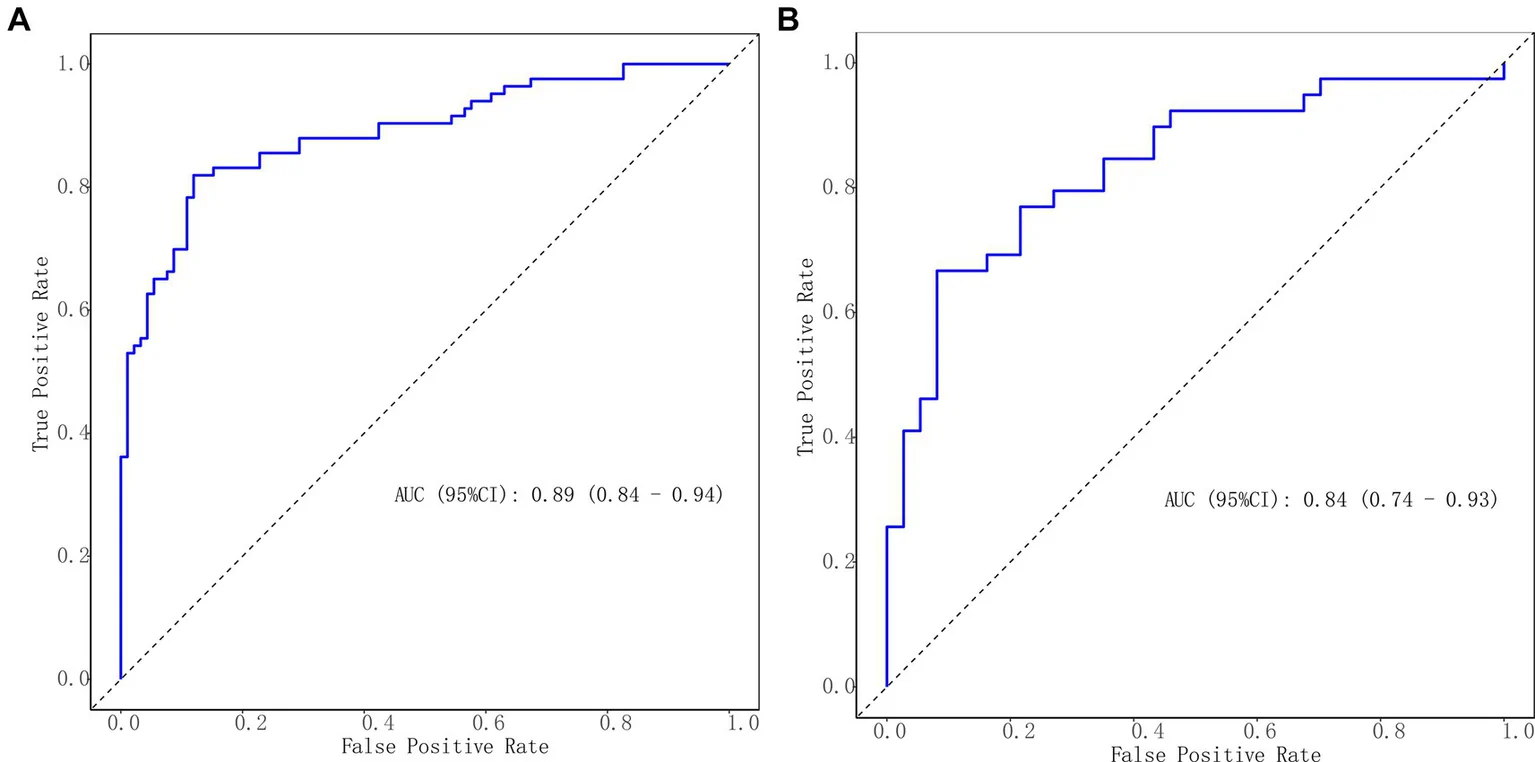
ROC curves of the nomogram prediction model for post-sarcopenia, with the x-axis representing specificity and the y-axis representing sensitivity. **(A)** ROC curve for the training set. **(B)** ROC curve for the validation set.

The discriminative ability of the risk score was analyzed by plotting ROC curves and calculating the AUC (Figure 3). The risk score demonstrated an AUC of 0.89 (95% CI: 0.84–0.94) in the training cohort and 0.84 (95% CI: 0.74–0.93) in the validation cohort, indicating its efficacy in distinguishing individuals at risk of post-sarcopenia. We used the ROC curve to identify a candidate cut-off of pt. ≈ 0.48 based on the maximum Youden index. At this threshold, accuracy was 0.85 (95% CI: 0.79–0.90), sensitivity 0.88 (95% CI: 0.81–0.95), specificity 0.82 (95% CI: 0.74–0.90), PPV 0.84 (95% CI: 0.77–0.92) and NPV 0.86 (95% CI: 0.78–0.94).

#### Calibration curve

**Figure 4:**
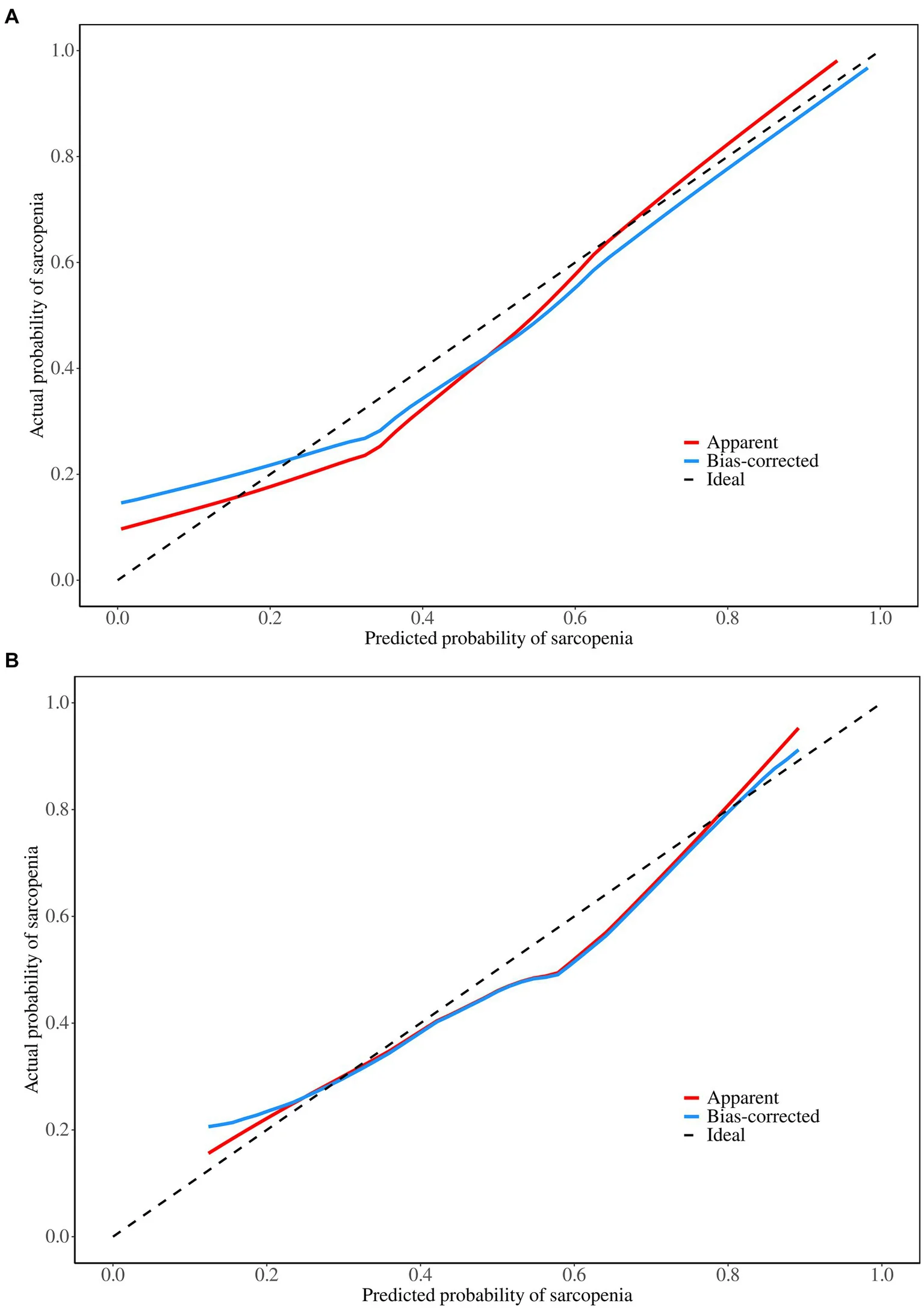
Calibration curves of the nomogram in the training and validation cohorts. **(A)** Training cohort. The apparent and bias-corrected calibration curves are plotted against the ideal 45° reference line, showing good agreement between predicted and observed probabilities (calibration intercept −0.07, slope 0.79, Brier score 0.003). **(B)** Validation cohort. The apparent and bias-corrected calibration curves similarly demonstrate acceptable agreement with the ideal line. In both cohorts, the Hosmer–Lemeshow goodness-of-fit test was non-significant (all *p* > 0.05), indicating no meaningful deviation from an ideal fit.

The nomogram’s calibration was assessed through calibration plots, the Hosmer-Lemeshow goodness-of-fit test, and numerical calibration indices. In the training cohort, the bias-corrected calibration curve showed strong alignment between predicted and observed probabilities (Figure 4). The calibration intercept and slope were −0.07 and 0.79, respectively, with a Brier score of 0.003, suggesting minimal systematic miscalibration and a small average prediction error. The Hosmer-Lemeshow test results were non-significant in both the training and validation cohorts (all *p* > 0.05), suggesting no meaningful departure from an ideal fit and supporting overall good calibration of the risk score.

#### Decision curve

**Figure 5:**
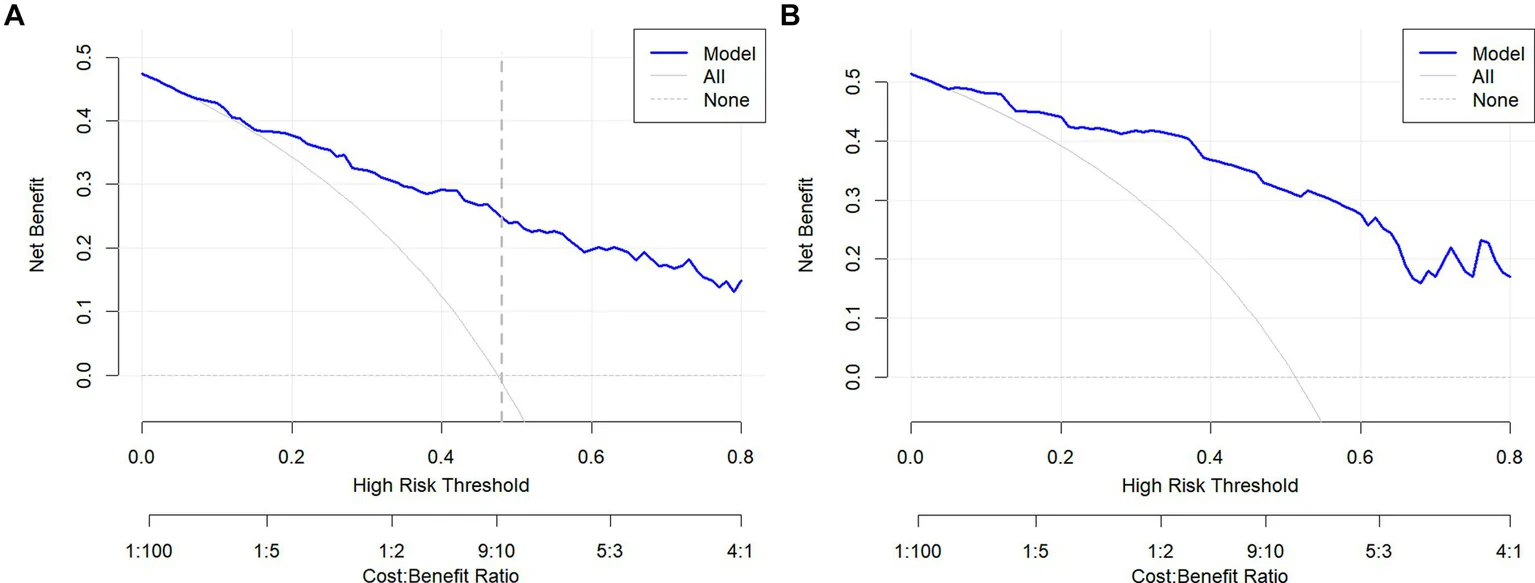
DCA curves for the nomogram prediction model of post-sarcopenia. **(A)** Training cohort. In decision curve analysis across threshold probabilities of 0–0.80, the nomogram consistently demonstrated positive net benefit compared with the “treat-all” and “treat-none” strategies. At the ROC-derived threshold (pt ≈ 0.48), the net benefit was 0.25 (95% CI: 0.16–0.35). Bootstrap resampling (*B* = 1,000) was used to obtain confidence intervals. **(B)** Validation cohort. The y-axis shows the net benefit and the x-axis the threshold probability. The blue solid line signifies model net benefit; the gray and black lines represent “treat-all” and “treat-none,” respectively.

Clinical benefit was assessed using decision curve analysis (DCA). In the clinically relevant region that includes pt. ≈ 0.48, the model’s net benefit curve remains above both the “treat-all” and “treat-none” strategies, indicating that using this risk threshold to trigger intensified follow-up would yield more true-positive cases without an excessive increase in unnecessary interventions (Figure 5).

## Discussion

Our study demonstrated that the prevalence of post-sarcopenia in early-stage NSCLC patients was 48.2%, while previous studies reported a prevalence of sarcopenia in lung cancer patients ranging from 9–56% (7). A novel screening model utilizing six accessible variables (gender, BMI, surgical method, PAL, postoperative albumin, and CRP) has been developed and validated to detect post-sarcopenia in early-stage NSCLC patients. Our findings indicated that this model was an effective screening tool for detecting muscle loss among early-stage NSCLC patients. Internal validation demonstrated the model’s robust screening capability, indicating its broad applicability for detecting post-sarcopenia. DCA revealed that the model conferred a net clinical benefit. To our knowledge, this is one of the first report of a risk score using readily available clinical data to identify early-stage NSCLC patients at risk for post-sarcopenia, thereby addressing a critical gap in risk stratification tools for this clinical context.

Currently, there is no consensus on the optimal method for assessing skeletal muscle mass. Common modalities include CT, MRI, DXA, BIA, and muscle ultrasound, each presenting distinct advantages and limitations. The predictive model employing CT to assess the SMI at the T12 level in this study offers significant advantages: low cost, objective evaluation data, reliable results, high reproducibility, and strong generalizability. Although standardized SMI cutoff values on CT are currently lacking, the T12-level threshold applied in our study has been validated in two independent investigations and demonstrated robust performance in diagnosing postoperative sarcopenia (11, 15). Crucially, compared to the commonly used L3 level, T12 measurement leverages routinely acquired preoperative thoracic CT imaging data, eliminating the need for additional abdominopelvic scans and thereby reducing both radiation exposure and associated costs.

Gender emerged as a pivotal determinant of sarcopenia risk in our cohort. Consistent with previous reports (13), our findings demonstrate that men with early-stage NSCLC are at a significantly higher risk of postoperative sarcopenia than women. Mechanistically, this male-specific susceptibility may be driven by the synergy between age-related muscle attrition and the acute suppression of the anabolic environment following surgery. The lower prevalence of post-sarcopenia observed in women suggests a protective physiological framework, albeit one with distinct etiological nuances. Estrogen appears to play a multifaceted role in preserving lean body mass and mitigating insulin resistance, particularly through the promotion of subcutaneous fat over metabolically deleterious visceral fat (16). Furthermore, female skeletal muscle is characterized by a higher density of Type 1 fibers and superior mitochondrial content, enhancing endurance capacity and fatty acid oxidation (17).

Furthermore, our nomogram included BMI as a variable for detecting postoperative sarcopenia, consistent with other studies (18, 19). The correlation between BMI and sarcopenia remains subject to debate. Previous reports have illustrated that low BMI may also pose a risk for sarcopenia, as it is often linked to reduced muscle mass, consistent with our findings (19, 20). Low BMI often reflects inadequate adipose and protein reserves, where limited amino acid availability suppresses the mTOR signaling pathway, thereby reducing muscle protein synthesis (20). Concurrently, malnutrition activates the ubiquitin-proteasome system, thereby accelerating muscle atrophy (21).

In the treatment of early-stage NSCLC, with the advancement of minimally invasive technology, the choice of surgical method has a significant impact on postoperative recovery and survival rates. In recent years, lobectomy has been widely regarded as the standard treatment for early-stage NSCLC, while sublobar resection (such as segmentectomy and wedge resection) has been increasingly recognized as an alternative approach (22). Studies have shown that different surgical methods may have varying effects on the prevalence of postoperative sarcopenia (23, 24). Our research identified sublobar resection as a predictive factor for postoperative sarcopenia in early-stage NSCLC patients. Specifically, compared to those of the lobectomy group, patients undergoing sublobar resection exhibited lower intraoperative blood loss and surgical stress responses such as metabolic and physiological disturbances, thereby lowering the risk of inflammation and metabolic exacerbation and reducing muscle loss (25). Moreover, patients undergoing sublobar resection exhibited higher postoperative albumin levels, indicating more stable protein metabolism due to surgical stress responses (26). Besides, Isaka et al. showed that postoperative psoas muscle loss was significantly less in patients undergoing segmentectomy than in those undergoing lobectomy (23). To date, there has been little research explaining the low invasiveness of segmentectomy from a physiological perspective. Research indicates that segmentectomy, compared with lobectomy, leads to reduced postoperative complications and improved physical, emotional, and cognitive functions, along with milder symptoms of dyspnea and fatigue 12 months after surgery (27). Extensive pulmonary resection not only exacerbates postoperative inflammatory responses but may also alter respiratory muscle loading by modifying neuromuscular interactions in the surgical field, thereby inducing both adaptive muscular remodeling and disuse atrophy (28). Therefore, our study substantiates that sublobar resection may be more favorable for preserving muscle mass in the early treatment of NSCLC, whereas patients undergoing lobectomy may require more attention during postoperative rehabilitation to reduce the risk of post-sarcopenia.

PAL is a frequent complication following lung resection, leading to prolonged chest tube placement, increased postoperative pain and prolonged hospital stay (14, 29). Although the direct impact of PAL on muscle loss has not been widely reported, our findings demonstrated that PAL represents a significant risk factor for developing post-sarcopenia in early-stage NSCLC patients. After surgery, patients may be restricted in their activities due to symptoms like difficulty breathing and pneumothorax. PAL may also induce persistent impairment of respiratory function and pain, thereby reducing postoperative physical activity (14). This activity limitation, especially with prolonged chest tube drainage, leads to prolonged bed rest, accelerating muscle atrophy. Furthermore, PAL and related respiratory discomfort increase the body’s stress response, disrupting metabolic balance and promoting muscle protein breakdown (30). Therefore, effective postoperative management and early activity interventions are crucial for preventing the development of post-sarcopenia. These include optimizing surgical techniques to reduce the risk of air leakage, as well as implementing respiratory and physical therapy in the early postoperative period to help patients resume normal activities as soon as possible and reduce muscle loss. These strategies not only improve the overall recovery outcomes for patients but also shorten hospital stays and lower medical costs.

Two laboratory markers, including CRP and postoperative albumin, were identified as independent predictors of post-sarcopenia. Systemic inflammation is another significant feature of cancer, promoting catabolism and contributing to body depletion. Inflammatory cytokines such as CRP, IL-6, and TNF-*α* are crucial for predicting sarcopenia. Previous studies have found that elevated CRP levels are associated with reduced muscle mass. A study of 3,369 men aged 40–79 studied the association between inflammatory markers and sarcopenia risk factors, but found no correlation between hs-CRP levels and muscle mass reduction (31). Overall, the association between elevated CRP levels and muscle mass remains unproven. Emphasizing the pathophysiological mechanisms leading to sarcopenia is crucial, especially before significant muscle function loss. Accumulating evidence has shown that systemic inflammation, mediated by markers such as CRP, is crucial in the occurrence and development of muscle degeneration and dysfunction (32). As an acute-phase inflammatory marker, CRP indicates heightened systemic inflammation following surgery. Further research is warranted to fully elucidate how CRP influences skeletal muscle and to identify therapeutic targets that counteract its negative impacts (33).

Serum albumin is a crucial marker for assessing nutritional status and has been recognized in prior research as an important prognostic factor for lung cancer outcomes following surgery (34). Low albumin is typically associated with chronic or acute inflammation (such as post-surgery or trauma), and inflammatory cytokines exacerbate muscle protein breakdown while inhibiting muscle protein synthesis, leading to slower muscle recovery (35). Postoperative hypoalbuminemia can increase capillary permeability, contributing to myocyte edema and protein loss (36). When albumin levels are low, the body’s repair capacity weakens, and muscle recovery and growth are inhibited. Our study emphasizes the adverse effects of low postoperative albumin levels on the prevalence of post-sarcopenia in early-stage NSCLC patients.

Our study has several limitations. Although clinically relevant factors were retained where possible and bootstrap internal validation was used to estimate optimism, model instability and overfitting cannot be excluded given the retrospective design and small internal validation cohort. In addition, this was a single-centre retrospective study including only patients with complete thoracic CT data, and the cohort was predominantly female and Chinese, which may limit generalizability to other populations, institutions, and pathological subtypes. External validation in larger multicentre cohorts is therefore warranted. Moreover, the cohort was limited to patients undergoing video-assisted thoracoscopic surgery, and patients who received preoperative chemotherapy were excluded; thus, applicability to thoracotomy, other surgical approaches, or pretreated patients remains uncertain. Future studies should incorporate EWGSOP2-based criteria, functional measures such as handgrip strength and gait speed, and prospective multicentre validation in broader surgical populations. Additionally, preoperative psychological status, dietary patterns, nutritional or exercise interventions, and serial CT assessments at earlier postoperative time points were not available in this retrospective cohort, which should be addressed in future prospective investigations.

## Conclusion

Overall, we successfully established and validated a risk score to identify early-stage NSCLC patients at risk for post-sarcopenia. This risk score serves as a valuable tool for clinicians to enhance sarcopenia screening and facilitate the implementation of targeted interventions to improve patient prognosis.

## Data Availability

The raw data supporting the conclusions of this article will be made available by the authors, without undue reservation.

## Footnotes

1
http://www.zstats.net
